# Observational evidence of the association between physical and psychological determinants of aging with cognition in older adults

**DOI:** 10.1038/s41598-024-58497-7

**Published:** 2024-05-31

**Authors:** Valéria Feijó Martins, Leonardo Alexandre Peyré-Tartaruga, Aline Nogueira Haas, Ana Carolina Kanitz, Flávia Gomes Martinez, Andréa Kruger Gonçalves

**Affiliations:** 1 Programa de Pós-Graduação em Ciências do Movimento Humano, Laboratório de Biodinâmica, Centro de Referência do Envelhecimento e Movimento, Universidade Federal do Rio Grande do Sul, Porto Alegre, Brazil; 2 Human Locomotion Laboratory (LocoLab), Department of Public Health, Experimental Medicine and Forensic Sciences, University of Pavia, Pavia, Italy; 3 Global Atlantic Fellow for Equity in Brain Health, Global Brain Health Institute, Trinity College Dublin, Dublin, Ireland

**Keywords:** Ageing, Geriatrics

## Abstract

Aging involves physical and cognitive deterioration in older adults. Detecting predictors of dementia early is crucial to identify its onset. This study aimed to associate physical and psychological determinants with cognitive performance in older adults. Observational study with 221 older adults, classified according to cognitive impairment. We evaluated cognitive function by Mini-Mental State Examination. Physical determinants encompassed muscle strength, functional mobility, and cardiorespiratory fitness, while psychological consisted of quality of life and activities of daily living. Multiple linear regression was performed to investigate this relationship. Physical and psychological determinants were significantly linked to cognitive impairment, including lower muscle strength, reduced functional mobility and of cardiorespiratory fitness. The influence of environment, autonomy, and engagement in daily activities on cognitive impairment was also observed. The analysis of physical and psychological determinants could help to aid in distinguishing individuals with cognitive impairment.

## Introduction

The global aging population has increased degenerative diseases occurrence and prevalence^1^. Over 50 million people worldwide are affected by dementia, which is expected to triple in the coming decades^2^. In Brazil, there is a high incidence of Alzheimer’s disease, particularly among white women aged over 70 years old^3^. However, a transitional stage exists before dementia diagnosis, involving older adults with cognitive impairments but no clear dementia symptoms. Understanding the behavior of people with and without cognitive impairment helps early detection of dementia predictors^4^ is crucial for delaying or identifying its onset condition.

Several studies depict the aging process as a biological phenomenon associated with physical function decline in older adults. These changes impact individuals’ interaction with their environment, ranging from basic tasks to more complex activities like object manipulation and walking^1,5,6^. Cognitive changes are an age-related phenomenon that affects attention, processing speed, reaction time, language, and executive function for both men and women^1,7^. It is a multidimensional process, with age being the primary risk factor, but other factors contribute to the progression of degenerative processes^8^. Studies suggest that different cognitive domains exhibit varying rates of decline, with verbal and numerical abilities tending to remain stable while memory and processing speed experience a greater decline^8^. Moreover, these cognitive changes are associated with structural alterations in the brain, including gray and white matter shrinkage^9^. Age-related changes in cognitive and physical function are linked to higher risks of disability, mortality, and loss of autonomy and quality of life^1,10,11^.

Muscle strength is an indicator of physical function, exhibiting age-related changes and decline over time^11,12^. Evidence shows an association between muscle strength and cognitive performance, demonstrating differences in motor behavior between older adults with and without cognitive impairment^10,13^. Evidence also suggests that muscle strength, given by grip strength, has prognostic value on the aging process representing the individual’s ability to execute motor commands, motor speed skills, and memory allocation. These adaptations seem sensitive to changes in brain health^11,13,14^.

Indeed, there is an association between functional mobility and cognitive measures in healthy older adults^15–17^. Evidence suggests that a decline in functional mobility, assessed by the timed up-and-go test, may be linked to an increased risk of cognitive function deficit and can differentiate older adults with mild cognitive impairment from those cognitively healthy. Consequently, older adults with better mobility tend to perform better on global cognition, executive function, and memory assessments^15,18^. Conversely, cognitive decline can negatively impact functional mobility, reducing the ability to perform complex movement tasks^19^. Hence, it is crucial to maintain both cognitive and physical health to prevent mobility issues and enhance the quality of life among older adults.

Regular engagement in aerobic exercise improves cognitive function^20,21^. This cognitive enhancement is attributed to the increased cerebral blood flow, facilitating oxygenation and nourishment of brain cells^22^. Furthermore, research suggests that cardiorespiratory fitness may be related to a reduced risk of age-related cognitive decline and neurodegenerative diseases such as Alzheimer’s disease, as older adults with better cardiorespiratory fitness demonstrated a lower likelihood of developing dementia over time^22,23^.

Quality of life and activities of daily living (ADL) indicate a person’s health status^24,25^. Cognitive deficits in memory in older adults are associated with difficulties in planning and frequency social activities, impaired communication, a higher likelihood of physical decline, a negative perception of health status, and worse quality of life^26–28^. The assessment instruments most frequently utilized rely on self-perception, and ADL have demonstrated associations with various cognitive measures in older adults. Furthermore, cognitive decline affects the performance of daily activities, resulting in impaired daily functions. In addition, ADLs such as medication responsibility, shopping, and financial handling were considered indicators for discriminating cognitive impairment^24^.

Although there are associations with some variables related to physical fitness and cognitive measures, this relationship is not yet well understood when evaluating different physical and psychological parameters.

Thus, this study aimed to associate possible physical and psychological parameters with cognitive functioning in older adults. We hypothesize that physical determinants (muscular strength, functional mobility, and cardiorespiratory fitness) would be positively associated with better cognitive functioning, while the psychological impairment (quality of life and activities of daily living) may contribute to the cognitive impairment detection. Further, we aimed to investigate the relationship between age and cognition and compare older adults with cognitive impairment versus older adults without cognitive impairment. The possible determinants of the differences in age-related cognitive decline are not fully understood. Thus, we also hypothesize that there is a direct negative association between age and cognition. It is important to emphasize that while association measures are valuable, they do not inherently establish a cause-and-effect relationship. However, the findings of this study would provide more evidence about the importance of preserving physical and psychological determinants of aging and their role in maintaining cognitive function, consequently promoting successful aging. These findings should contribute to the organization of mental and exercise concurrent interventions.

## Methods

### Study design and participants

This investigation is an observational study analyzing the baseline data from the database of a controlled, randomized, double-blind, stepped wedge and interdisciplinary clinical trial available in the Reference Center for Aging and Movement (CREM), programme at the School of Physical Education, Physiotherapy and Dance, of Federal University of Rio Grande do Sul (UFRGS), Brazil. The CREM programme aims to be a reference in the study of movement and aging, delivering different physical exercise modalities and developing research about its effects on clinical and functional outcomes in older adults. This study was approved by the Research Ethics Committee of the Federal University of Rio Grande do Sul (CEP-UFRGS; n65435022.9.0000.5347). The procedures conformed to the latest revision of the Declaration of Helsinki. Participants were previously informed about the research and the free and informed consent term was read and signed.

The participants are older adults from the Brazilian South region recruited from the CREM’s database. The participant’s inclusion criteria were: be over 60 years old, be literate, have a medical authorization to practice exercises and ability to walk independently. Exclusion criteria included: not showing medical clearance to practice exercise; presenting medical comorbidities and/or medical conditions that contradicted participation in the study; history of physical-cognitive impairment; did not participate in all evaluations. Consequently, the sample consisted of 221 older adults from the database eligible to participate in the study, divided into two groups, 174 subjects were classified without cognitive impairment and 47 with cognitive impairment.

We assessed cognitive function by Mini-Mental state examination^29,30^. This test assesses cognitive functions, time and location orientation, word registration and recall, attention and calculation, language, and visual constructive ability^31^. Higher test scores (maximum 30 points) represent better cognitive performance. This instrument was employed for sample characterization with the aim of identifying cognitive declines for group categorization and the cutoff point for cognition was according to years of formal education and the achieved score: 1–5 years of schooling < 24 points; 6–11 years < 26 points; ≥ 12 years < 27 points^31^. From this, 78% of the participants do not have cognitive impairment, with 22% presenting cognitive impairment.

### Assessment tools and outcomes

The participant’s age (years), sex, ethnicity (race), socioeconomic status (self-declaration of family income), and education level (school qualification) was assessed using an anamnesis. Age groups were classified per decade into 60–69, 70–79, and ≥ 80 years of age to distinguish potential changes that may occur with the aging process. All the instruments used to collect cognitive function, physical determinants of aging, ADL and quality of life are considered the gold standard for the study population. All assessments were carried out by experienced researchers.

The physical determinants of aging included muscle strength, functional mobility, and cardiorespiratory fitness. The muscle strength was assessed using the five-repetition sit-to-stand test^32,33^, measuring the time taken (in seconds) for the participant to complete the action of getting up from a chair and then sitting down five times as quickly as possible. The “Timed Up and Go” (TUG) test was used to assess functional mobility, measuring the time (in seconds) that the participant takes to get up from a chair, walk 3 m, go around a cone, walk back and sit down^34,35^. The 6 min walk test (6MWT) was used to assess cardiorespiratory fitness. The participant was asked to walk as far as possible during this test for 6 min. The distance covered was used to evaluate exercise tolerance and functional capacity, indicating the participant’s physical condition and monitoring changes over time^36,37^.

The advanced activities of daily living (ADL) scale was used to assess the ability to perform specific tasks, such as managing finances, preparing meals, shopping, using transportation, and carrying out household chores^38,39^. The scores were classified according to participation in ADL, with the most active performing four or more activities and the least active performing three or fewer activities^40,41^. A higher score indicates that participants are more active, while a lower score indicates greater dependence and possible need for assistance.

The World Health Organization Quality of Life questionnaire—brief Brazilian version (WHOQOL-Bref) was used to assess the quality of life^42,43^. This questionnaire consists of 26 questions covering the following of four domains: physical, psychological, social relations and environment. The WHOQOL-Old^44,45^ was also used to assess the quality of life, an instrument that evaluates the quality of life in aging-related issues. This questionnaire is divided into six domains: sensory functioning; autonomy; past, present and future activities; social participation; death and dying; and intimacy.

The dataset generated during the current study is available in the Figshare repository, 10.6084/m9.figshare.25347868.

### Statistical analysis

We used absolute and relative (%) frequencies for categoric variables, and mean and standard deviation for continuous variables. Descriptive data are presented according to the presence of cognitive impairment (group with no cognitive impairment and group with cognitive impairment).

Multiple linear regression analyses with the enter method were performed to investigate the relationship between physical and psychological determinants and cognitive function. Adjustments for cognitive function by education were proposed by Duncan^31^. Muscle strength, functional mobility, cardiorespiratory fitness, and quality of life were considered continuous variables, while cognitive function, activities of daily living, and age were treated as categorical variables. All statistical analyses were performed using JASP for MacOS version 0.17.1 (https://jasp-stats.org/, University Amsterdam). Results from the logistic and linear regression model are presented as the odds ratios (ORs), 95% confidence intervals and p values.

## Results

The sociodemographic characteristics, physical and psychological determinants are stratified by group (with and without cognitive impairment) and are summarized in the Table 1. Most participants without cognitive impairment were women who were between 70 and 79 years old (48%). These were mainly white (89%), married (45%), income from 1 to 3 (33%) and from 4 to 6 minimum Brazilian wages (32%), university level of education (67%) and 96% more active. Those with cognitive impairment showed the same socioeconomic characteristics (85% women, 63% aged 70–79 years, 31% married, 33% income from 1 to 3 and 29% 4 to 6 minimum Brazilian wages, 38% university level of education) and 83% more active. There were differences between groups in the muscle strength (p = 0.036), functional mobility (p = 0.009), cardiorespiratory fitness (p = 0.010) and quality of life (environment domain p = 0.003 from WHOQOL-Bref and autonomy domain p < 0.001 from WHOQOL-Old).

**Table 1:** Description of sociodemographic characteristics, physical and psychological determinants of older adults with and without cognitive impairment.

		No cognitive impairment (n=174)	With cognitive impairment (n=47)
		Percent or mean SD	Percent or mean SD
Sex	Feminine	84	85
	Masculine	16	15
Races*	Black	7	24
	White	89	71
	Brown	4	5
Marital status*	Single	15	17
	Married	45	31
	Divorced/separated	21	24
	Widowed	19	28
Education level*	4-7 years	4	21
	8-11 years	27	36
	≥12 years	67	38
	1-3 years	2	5
Socioeconomic status*	≤1 MW	9	24
	1-3 MW	33	33
	4-6 MW	32	29
	≥7 MW	15	5
Age group	60-69 years	35	19
	70-79 years	48	63
	≥80 years	17	17
Activities of daily living*	More active	96	83
	Less active	4	17
Cognitive function		28.5 ± 1.3	24.3 ± 2.2
Muscle strength*		10.2 ± 3.2	11.7 ± 5.6
Functional mobility		6.56 ± 1.39	7.33 ± 2.64
Functional exercise capacity		480 ± 90.9	440 ± 92.3
Quality of life - physical		73.3 ± 15.3	72.1 ± 13.9
Quality of life - psychological		64.2 ± 9.9	60.8 ± 9.0
Quality of life - social relations		72.5 ± 15.9	72.2 ± 11.1
Quality of life - environment		73.6 ± 13.6	66.8 ± 11.5
Quality of life old - sensory functioning		30.8 ± 11.5	29.2 ± 11.4
Quality of life old - autonomy		76.1 ± 12.9	68.1 ± 15.9
Quality of life old - past, present and future activities		72.9 ± 13.2	70.7 ± 14.4
Quality of life old - social participation		69.9 ± 15.1	67.1 ± 15.3
Quality of life old - death and dying		71.0 ± 23.4	73.7 ± 21.2
Quality of life old - intimacy		74.7 ± 14.6	70.6 ± 16.8

Muscle strength and functional mobility in seconds; cardiorespiratory fitness in meters; quality of life in punctuation.

*MBW* minimum Brazilian wage in the moment the study period $229.00, *Mean SD* mean and standard deviation.

*The participant abstains from answering.

**Figure 1:**
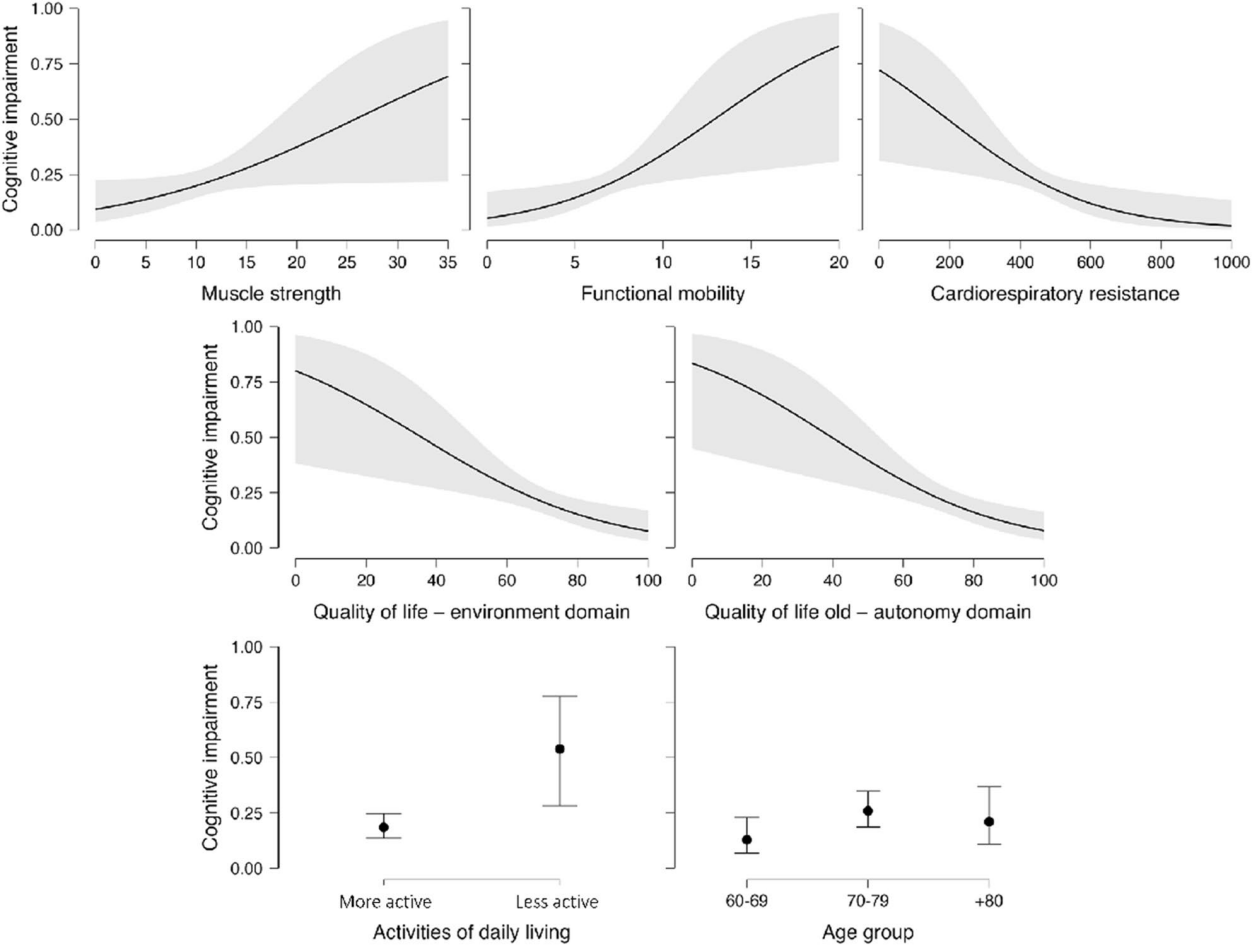
Representative graphs of the logistic regression model for cognitive impairment and physical and psychological determinants of aging.

Table 2 summarizes statistically significant results from the logistic regression for all participants. Muscle strength (OR = 1.09; p = 0.045), functional mobility (OR = 1.18; p = 0.042), cardiorespiratory fitness (OR = 0.99; p = 0.022), age group (OR = 2.37; p = 0.039), quality of life environment domain (OR = 0.95; p = 0.006) and autonomy domain (OR = 0.95; p = 0.002), and activities of daily living (OR = 5.12; p = 0.005) were significantly associated with cognitive impairment. Cognitive impairment classification (Fig. 1) was associated with lower muscle strength, reduced functional mobility and lower functional exercise capacity. The environment and autonomy seem to influence cognitive impairment, as well as involvement in activities of daily living. In this sample, according to Table 2, individuals aged 80 and older have better cognitive function compared to individuals in the 70–79 age group.

**Table 2:** Logistic regression model for cognitive impairment and physical and psychological determinants of aging.

	Odds ratio	95% CI lower bound	95% CI upper bound	Standard error	Sig
Muscle strength	1.092	0.002	0.174	0.044	0.045
Functional mobility	1.178	− 4.213	− 1.568	0.081	0.042
Cardiorespiratory fitness	0.996	− 0.009	− 0.001	0.002	0.022
Quality of life-environment domain	0.949	− 0.065	− 0.012	0.019	0.006
Quality of life old-autonomy domain	0.953	− 0.066	− 0.015	0.013	0.002
Activities of daily living	5.133	0.485	2.786	0.587	0.005
Age group	2.368	0.044	1.680	0.417	0.039

Cognitive impairment level ‘with cognitive impairment’ coded as class 1; the reference category for age group is 70–79 years; the reference category for activities of daily living is less active.

## Discussion

The present study aimed to examine the physical and psychological determinants of aging in relation to cognitive performance. Cognitive performance was associated with physical determinants of aging (muscle strength, functional mobility, and cardiorespiratory fitness, and psychological factors, including domains of autonomy and environment in quality of life, as well as activities of daily living). This association can contribute to determining cognitive performance, as higher scores were observed in older adults with normal cognitive function. Thus, our hypotheses were confirmed, revealing that older adults with cognitive impairment have lower muscle strength, diminished functional mobility, and reduced cardiorespiratory fitness. These factors significantly impact the performance activities of daily living and result in decreased quality of life domains such as environmental conditions and autonomy.

Systematic reviews have shown an association between lower grip strength and reduced cognitive function over time, indicating that greater handgrip strength exert a protective factor against cognitive decline^13,14,46^. Also, the handgrip strength represents a predictive factor not just to decline of cognition, as well as for mobility, functional status and mortality in older populations^14^. These findings suggest that strength may be linked to multiple aspects of cognitive function, providing a longer life for those who experience greater levels of strength^9,46^. However, findings on strength of the lower limb are lacking. We identified a study that linked memory capacity to lower limb strength, demonstrating that cognitive function can be associated with physical fitness^47^. A significant association was identified between the five-times-sit-to-stand test and cognitive performance, indicating that a performance duration of less than 15 s serves as a cutoff value for determining the presence of cognitive impairment^48^. Interestingly, our results were below this cutoff point suggested by the literature (10.2 ± 3.2 s for the group no cognitive impairment and 11.7 ± 5.6 s for the group with cognitive impairment). The aged people with cognitive impairment had a mean age of 74.1 ± 7.3 years, which was lower than the previous study (mean age 80.4 ± 0.04 years). The sample of this study consisted of older French individuals. Thus, further studies are needed to shed light to these differences taking into accounting social and health aspects. Other studies also indicate results below the cutoff point^49,50^. It should be noted that the results of functional tests may vary due to the characteristics of the sample (body composition, previous experience with physical exercise, people in real life conditions), study setting (from region to university or neighborhood), as well as the instructions given at the time of the assessment.

An association between cardiorespiratory fitness and cognitive function was found. The participants with lower cardiorespiratory fitness were higher in the group classified with cognitive impairment than those who had better cognition. These results were in line with previous studies that showed cardiorespiratory resistance was strongly associated with cognitive function, specifically attention^47,51^. Healthy older adults with higher cardiorespiratory fitness may be related to the effectiveness of the global brain network, resulting in an improved executive function^47,52^.

We also found an association between functional mobility and cognitive impairment, as lower scores in the TUG test were related to cognitive impairment. Corroborating our results, the TUG test could potentially help distinguish individuals with poor cognitive performance^53^. Previous studies investigating the relationship between cognitive function and functional mobility outcomes corroborate the findings of the present study^15,53^. The cognitive processes play a crucial role in adapting to a dynamic environment, enabling individuals to maintain postural control and motor coordination during functional mobility^15^.

The cognitive impairment was related to autonomy and environment domains of quality of life, while other domains were not related. What was an unexpected result, as we expected more associations with the group with cognitive impairment. But these found their autonomy and environment play a significant role in cognitive functioning, particularly at early stages of cognitive decline^54^. Needing care, being dependent on activities of daily living, experiencing pain, and loneliness were associated with reduced quality of life^55^. Research investigating the relationship between quality of life, level of dependency, and cognitive functioning demonstrates that in older adults residing in their own homes the level of dependency serves as a significant predictor for the physical and environmental domains of quality of life^25^.

Our findings regarding the relationship between age and cognition demonstrated that older adults in the age range of 70 years exhibited poorer cognitive performance compared to both younger and older individuals. This indicates a potential for cognitive decline in this age group, which is a speculation based on the studied sample and warrants further exploration in future studies. However, age-related changes in the brains of older adults are not specified in terms of a specific age. Brain and cognitive changes vary from person to person and are influenced by factors such as lifestyle, overall health, and genetic predisposition^56^.

Based on available studies, this is the first study to demonstrate the association between cognitive performance and physical determinants of aging (muscle strength, functional mobility, and cardiorespiratory resistance) and psychological determinants (activities of daily living and quality of life) in older adults. The studied outcomes can contribute to the identification of cognitive impairment. The main implications of the study findings can be translated into clinical practice, offering to healthcare professionals the opportunity to identify and address often overlooked issues by assessing the physical and psychological determinants of aging in older adults. This underscores the importance of a complementary and confirmatory approach to cognitive performance assessment.

The present study has some limitations. Firstly, although our cross-sectional study demonstrates a robust association between physical and psychological outcomes and cognitive capacity, these findings by no means infer causality. However, we present a rationale to support a plausible link between these factors, as determined in our study. Another limitation was our results are self-reported potentially suffering from social desirability bias, recall bias or response. The composition of the sample may be considered a limitation; we opted to utilize data from the community participants of the extension program, resulting in varying sample sizes and consequently non-matched groups. While we acknowledge the instruments limitations to assess cognition, the MMSE was used in this study, given its widespread usage in Brazil for cognitive screening and evaluation^57^.

Cognitive performance was associated with physical determinants of aging (muscle strength, functional mobility, and cardiorespiratory fitness), and psychological factors (quality of life and activities of daily living). Furthermore, the results showed an association between the age group of 70 years and the initial manifestation of cognitive function decline. Collectively, these results confirm our hypothesis and emphasize that cognitive function is associated with muscle strength, functional mobility, and cardiorespiratory fitness, as well the domains of autonomy and environment of quality of life and activities of daily living.

From a clinical perspective, our results also suggest that the five-times-sit-to-stand test, timed up and go test, and 6 min walk test could potentially help distinguish individuals with cognitive impairment. In this context, a healthcare professional specialized in working with older adults, particularly in the area of physical exercise, must be aware of older adults who perform poorly in physical tests in relation to their age group. This decrease in physical performance may serve as an indicator of potentially impaired cognitive function. However, it is important to highlight that the results may not be generalizable to all older populations, considering the cultural and social differences that can influence cognitive performance and the physical determinants of aging. A suggestion for future studies is to explore different strategies of physical training to promote positive adaptations concerning the interactions between physical function, clinical-functional aspects, and cognition.

## Data Availability

The dataset generated during the current study is available in the Figshare repository, 10.6084/m9.figshare.25347868.
